# Dosimetric Algorithm to Reproduce Isodose Curves Obtained from a LINAC

**DOI:** 10.1155/2014/849505

**Published:** 2014-06-18

**Authors:** Julio Cesar Estrada Espinosa, Segundo Agustín Martínez Ovalle, Cinthia Kotzian Pereira Benavides

**Affiliations:** ^1^Departamento de Radioterapia Oncológica, Hospital Universitario, Carretera Saltillo-Monterrey Km 4.5, 25204 Saltillo, COAH, Mexico; ^2^Grupo de Física Nuclear Aplicada y Simulación, Universidad Pedagógica y Tecnológica de Colombia, Avenida Central del Norte 39-115, Tunja 150003, Colombia; ^3^Departamento de Medicina Nuclear, Hospital Universitario, Carretera Saltillo-Monterrey Km 4.5, 25204 Saltillo, COAH, Mexico

## Abstract

In this work isodose curves are obtained by the use of a new dosimetric algorithm using numerical data from percentage depth dose (PDD) and the maximum absorbed dose profile, calculated by Monte Carlo in a 18 MV LINAC. The software allows reproducing the absorbed dose percentage in the whole irradiated volume quickly and with a good approximation. To validate results an 18 MV LINAC with a whole geometry and a water phantom were constructed. On this construction, the distinct simulations were processed by the MCNPX code and then obtained the PDD and profiles for the whole depths of the radiation beam. The results data were used by the code to produce the dose percentages in any point of the irradiated volume. The absorbed dose for any voxel's size was also reproduced at any point of the irradiated volume, even when the voxels are considered to be of a pixel's size. The dosimetric algorithm is able to reproduce the absorbed dose induced by a radiation beam over a water phantom, considering PDD and profiles, whose maximum percent value is in the build-up region. Calculation time for the algorithm is only a few seconds, compared with the days taken when it is carried out by Monte Carlo.

## 1. Introduction

The calibration of equipment generating ionizing radiation is a critical task that requires techniques, protocols, and established recommendations from international organizations. The latter are in charge of the correct application of the ionizing radiation in whole disciplines, as the ones involved with therapeutic cancer treatment. Megavoltage X-ray beams take into account deep penetration of different materials, also known as radiation quality [1]. One way to characterize the radiation beam is by the energy spectral distribution (difficult to measure), which is directly related to photons fluency distribution emitted by the LINAC [2]. Another method to solve this question is by using Monte Carlo techniques for calculating clinic radio therapeutic doses, where a good approximation is possible to obtain for photons spectra, electrons, or neutrons, all also produced by the LINAC [2, 3]. The results are very similar to those obtained by commercial planning systems [4–8].

The simulation to model treatments in teletherapy, is one of the most important aspects and of transcendency in therapeutic treatments against cancer. For decades there have been several attempts to systemize such processes. The first of them was used to model the contribution of the resultant dose of isodose curves from the contribution of several ionizing fields. Such systems were basically limited by the technology at such time [4]. In later years the trend has been to improve the accuracy in the calculations of the absorbed dose simulators implemented in reducing the computation time.

Following generation of planning systems allowed results integration obtained from the digital images procedures containing patient's anatomical structures. The following generation of planning systems allowed results integration obtained from the digital images procedures containing patient's anatomical structures, allowing the graphical superposition from the isodose curves on the tomographic images [1], through the knowledge of absorbed doses percentages related to deepness (PDD), as well as the corresponding profiles (PPD), for the establishment of a good estimation in the beam quality, and through the precise knowledge of such parameters [2, 3, 7, 9].

The dosimetric algorithm developed in this work calculates the dose percentage from the values obtained through the MCNPX 2.5 code [10]. It was used to simulate a linear accelerator Varian Clinac 2100C/D operating at 18 MV energy with dose calculation over a water phantom. The PDD and dose profiles were analyzed and compared with experimental data.

## 2. Materials and Methods

**Figure 1:**
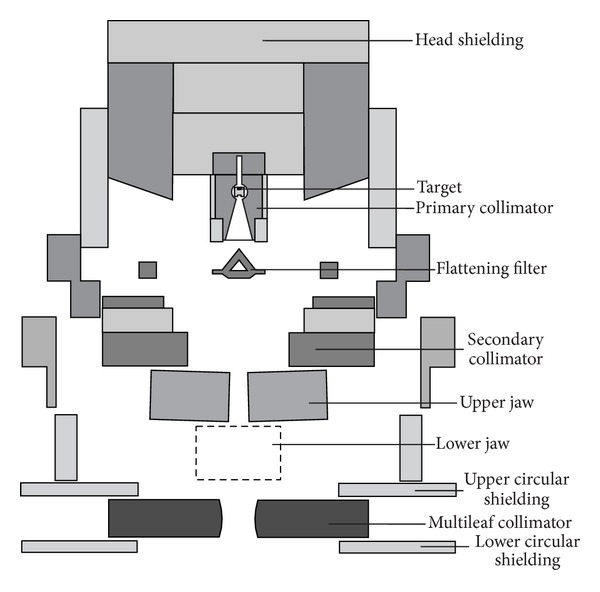
Geometry of the accelerator Varian Clinac 2100C/D, *YZ* plane. Table 1 of [15] shows the materials of the main components of the LINAC head.

The whole LINAC geometry was constructed following Varian's specifications.  Figure 1 shows the *YZ* plane geometry where the main components of the accelerator are illustrated; particular characteristics of such LINAC can be found elsewhere [11]. Phantom used for water calculations has the following dimensions: 50 × 50 ×  30 cm^3^ located at a 100 cm distance from the X-ray source. Such LINAC operates with dual energies 6 and 18 MV and is located at the Hospital Virgen de las Nieves in the city of Granada, Spain. Experimental data used for the dose profile of synchronization energy and the PDD were taken at the Hospital's LINAC.

**Figure 2:**
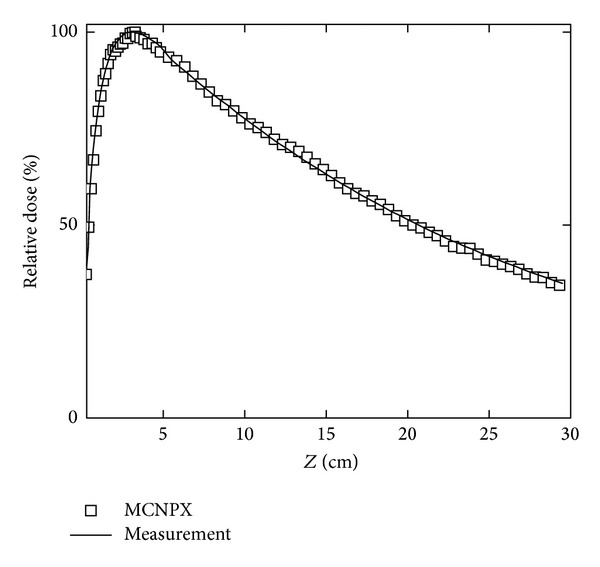
Calculated and measured PDDs in water for the LINAC used in this work. Experimental data were taken from [16].

**Figure 3:**
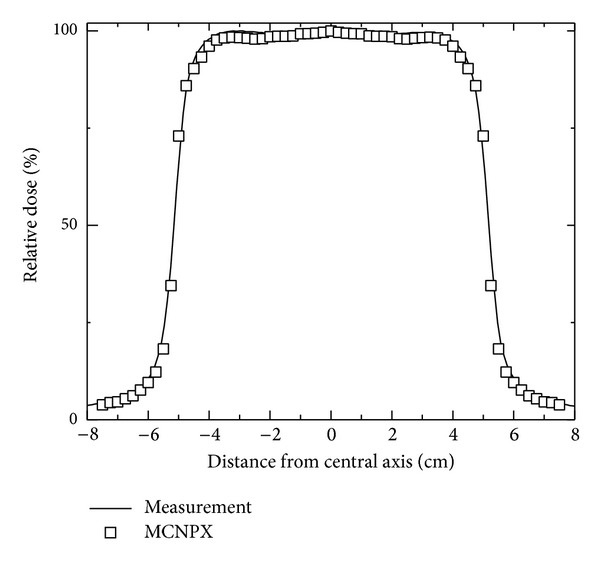
Calculated and measured dose profiles at 3.25 cm depth for the LINAC.

Collimators and multileaf collimator systems were adjusted to a 10 × 10 cm^2^ square field. Electrons of 18.02 MeV were impacted over the target after the synchronization process was carried out, where the experimental and calculated PDDs were adjusted, as shown in Figure 2. The PDD value was measured and calculated over the beam axis at 3.25 cm of depth in the build-up region; the maximum dose value given by the LINAC corresponds to 6.57 × 10^−16^ Gy *·* e^−1^. At such depth the corresponding dose profile calculated was also shown with the experimental measure (Figure 3). Both data groups were used by the implemented algorithm in this work to reproduce absorbed dose values at any point or voxel of the phantom.

The *F8/MCNPX tally estimates the absorbed dose calculations over 3360 voxels of 0.125 cm^3^ located over the beam axis in the whole plane that reproduces profiles. Such group of data gave a total of 56 absorbed dose profiles and their corresponding PDDs. Five independent calculations were necessary considering the amount of voxels used; it was executed under a Linux platform. A total of 9 × 10^9^ histories using 8 PC processors in parallel were carried out taking an approximate time of 8.5 days each. The energy cut-off for photons was established in 0.001 MeV for the whole geometry. For electrons 3 energies cut-offs were established: inside the LINAC's head and with the beam axis direction, it was 0.521 MeV; for the rest of the Linac head components, was established in 2 MeV, understanding that electrons do not escape from the region, and 0.001 MeV for all of the water phantom.

### 2.1. Algorithm's Generalities

The algorithm code was developed using the Visual Basic 6.0 programming language with a high level graphical interface executed through the Windows XP or superior operative system. The minimum hardware requirements for such applications are dual Pentium IV 3.4 GHz or superior microprocessor, 80 MB free space or more to save created image archives, graphic card 16 MB at least, high resolution SVGA monitor, and a video accelerator graphical card. Estimated time for dose percentage calculations in a pixel-to-pixel plane is of only few seconds.

### 2.2. Reconstruction Method

Dose percentage calculation methodologies were carried out through multiple interpolations, first taking the absorbed dose percentage values in the voxels of PDD located over the beam axis and below the phantom surface; in addition, the profile percentage values were the maximum dose registered under the simulation with MCNPX, as indicated in

(1) [VPPDiVPDDj], ∀{i,i=1,2,3,…,n,j,j=1,2,3,…,m.

**Figure 4:**
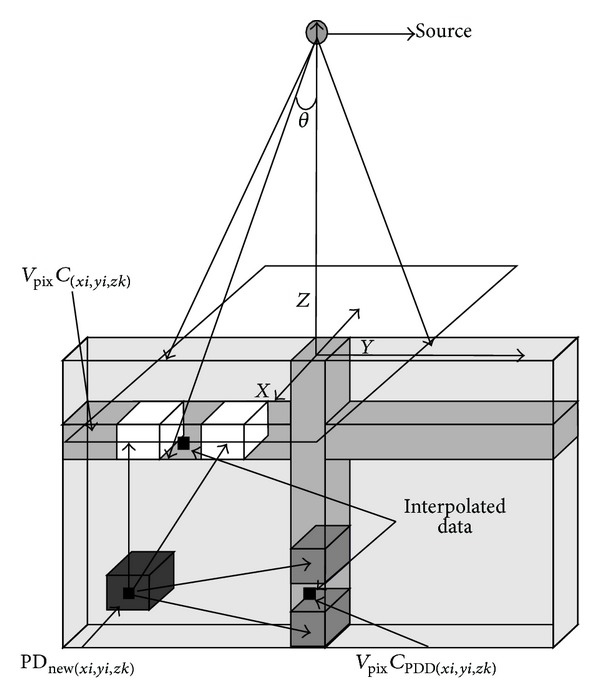
Unscaled scheme showing the beam divergence geometry to obtain the PD_new_ values in the whole plane. Values obtained from MCNPX simulation.

These values correspond to the ones observed in Figures 2 and 3, respectively. After that the new absorbed dose percentages were calculated through a pixel-to-pixel interpolation, now called (PD_new_). It was necessary to calculate the *V*_pix_ *C*_PDD_*j*__ values from the lineal interpolation between pixels and the initial PDD voxel's values and the values *V*_pix_ *C*_PPD_*i*__ correspond to the pixels in the voxels of the absorbed dose profile, both previously obtained from the simulation with the MCNPX code shown in (2) and illustrated in Figure 4:

(2) [VpixCPPDjVpixCPDDk], ∀{j,j=1,2,3,…,n,k,k=1,2,3,…,m.

Finally we calculate the remaining PD_new_ values over the plane, interpolating as schematically illustrated in Figure 4, where the PD_new_ values are calculated with the dosimetric algorithm from the interpolated data *V*_pix_ *C*_PDD_*k*__ following the line of the divergent beam angle (*θ*), and the voxel value *V*_pix_ *C*_PDD_*k*__ corresponding to the depth (*k*), shown in Figure 4 as the smaller frames (interpolated data) *V*_pix_ *C*_PPD_*j*__ and *V*_pix_ *C*_PDD_*k*__, respectively; then we calculate the product between the voxels (*V*_pix_ *C*_PPD_*j*__ · *V*_pix_ *C*_PDD_*k*__), to construct the *I*(*i*, *j*) matrix [12] from (3). The PD_new_ value is represented in a darker tone in Figure 4 and is obtained from the different interpolations made using the *V*_PPD_*i*__ and *V*_PDD_*j*__ values initially given in the algorithm:

(3) ∑j=1m∑i=1nPDnew(i,j)=∑k=1m∑j=1n(VpixCPPDj)(VpixCPDDk)I(i,j)=(PDnew(1,1)⋯PDnew(1,j)⋮⋱⋮PDnew(i,1)⋯PDnew(i,j))n×m.

In the vertical axis from Figure 4 and over *Z* axis there are percentage PDD values, while in the horizontal axis there is the percentage of absorbed doses profile values, where the maximum dose deposit from the PDD is registered. This is located at 3.25 cm depth below the phantom surface. The 18.02 MeV electrons source is located at a 100 cm distance over the phantom surface. Finally in the *XY* plane the correspondent distribution belonging to the field size of 10 × 10 cm^2^ used for this simulation is shown.

**Figure 5:**
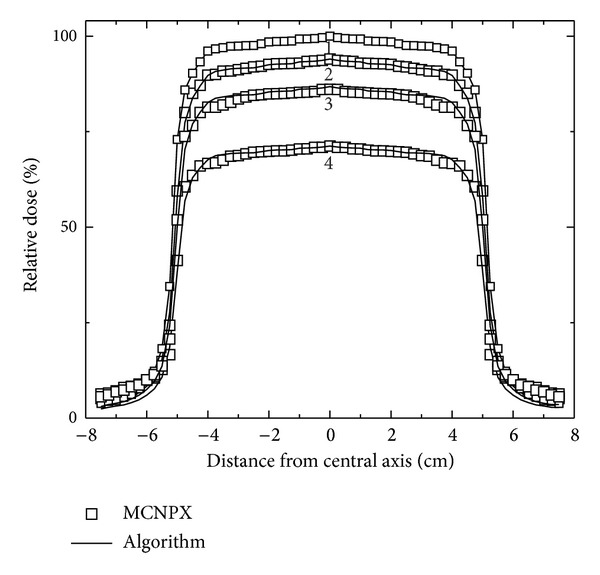
Absorbed dose profiles comparative up to the dose maximum (3.25 cm) in the build-up region.

The absorbed dose percentage values gave shape to the dose distribution in the plane, also represented in (3), and built a matrix with *n* × *m* dimensions. Four profiles from the matrix, in the build-up region at the depths of 0.75, 1.25, 1.75, and 3.25 cm, were selected and compared with the profiles of the simulation at the same depths. Results are illustrated in Figure 5 with the labels 4, 3, 2, and 1, respectively, where profile 1 is located at the 3.25 cm depth and used as a reference for the values calculation in the dosimetric algorithm.

**Figure 6:**
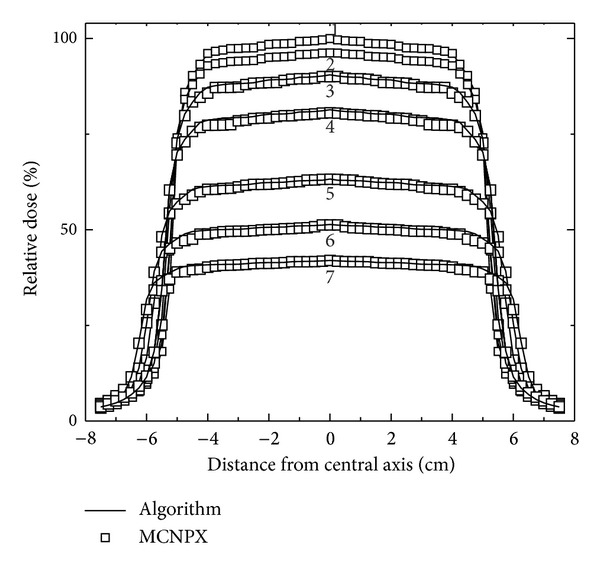
Comparison of absorbed dose profiles measured from the point of the maximum dose (3.25 cm) up to profile 7 (25.25 cm).

A second absorbed dose profile set was compared with the values obtained from the dosimetric algorithm (Figure 6). Seven profiles labeled from 1 to 7 were selected corresponding to the depth values of 3.25, 4.75, 6.75, 9.25, 15.25, 20.25, and 25.25 cm, respectively. The results shown in Figures 5 and 6 established the agreement when they are compared; these allow seeing the precision of the algorithm at the moment to obtain the absorbed dose percent values. There is a good approximation among the voxels where a radiation beam intersection exists with the water containing in the phantom, as well as optimizing calculation time.

The uncertainty in the calculations in the penumbra region, that is, outside the field of increases in the first cm depth, due to the interaction of the photon beam polyenergetic, generating an increase in scattered radiation; this causes discrepancy between the calculations by the two methods as shown in Figure 5. Furthermore, in Figure 6, the calculations in the penumbra are more consistent as far as depth increases, obtaining an absorbed dose more uniform by the electrons in the medium. However, the radiobiological effect on the penumbra is considered negligible.

### 2.3. Visual Reconstruction of PD**_ new_**

**Figure 7:**
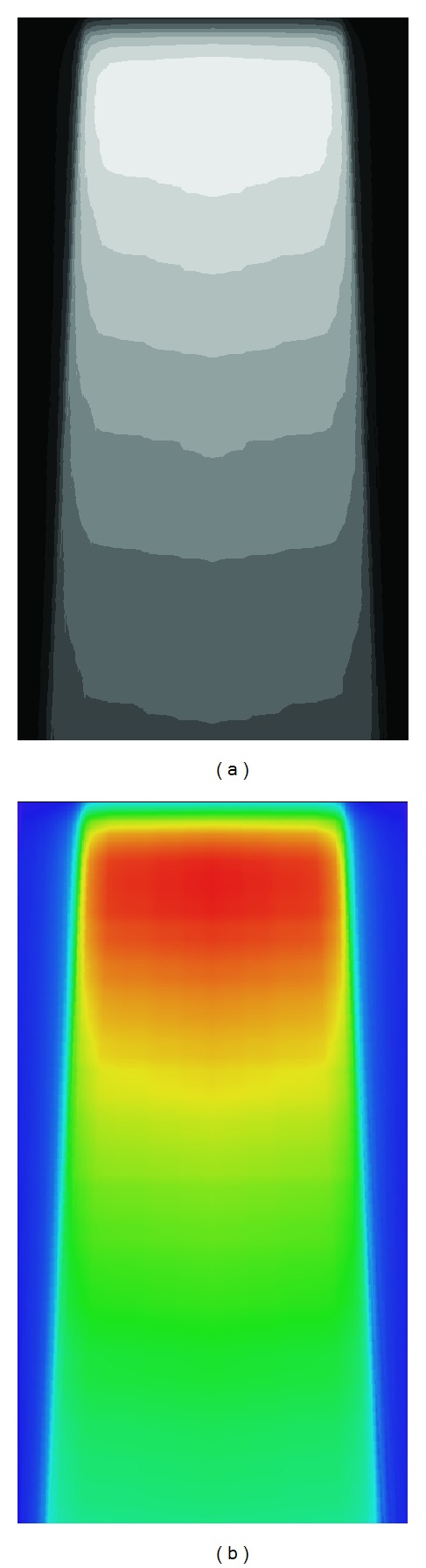
(a) Isodose curves at different depths shown in grey tones. In (b) representation of isodose now in the color space.

Once the absorbed dose percentage values (PD_new_) were obtained by means of the algorithm calculation, the visual reconstruction of the image associated with the values is carried out. The PD_new_ values were stored in specific files, as well as the values calculated by the Monte Carlo techniques, which were previously normalized with respect to the dose percent maximum located at the 3.25 cm depth below the water surface inside the phantom and over the radiation beam symmetry. Finally the image could be reconstructed and illustrated, at gray tones (Figure 7(a)) and colored (Figure 7(b)) by using the transformation scale HSL (hue, saturation, and lightness).

#### 2.3.1. Visualization of Results from Grey Scale Tones

Once the pixel-to-pixel percentage values were obtained, then the matrix from (3) was constructed. It required transformation from the absorbed dose percentage values to the corresponding grey tones by using (4). Each element of the matrix represents a pixel, and then an 8-bit image was constructed. Each element of the matrix was transformed into values within the 0–255 range, according to (4).

The first element of the matrix represents the indexed location of the pixel in the image within the plane, as well as three grey tone values associated in such a point for each of the 3 channels RGB (red, green, and blue) [13]:

(4) R(PDnew(i,j))=PDnew(i,j)·255,G(PDnew(i,j))=PDnew(i,j)·255,B(PDnew(i,j))=PDnew(i,j)·255.

The number 255 represents the maximum value of the grey scale tone, corresponding to the darker or lighter convenience. *R*, *G*, and *B* represents the values obtained within each of the 3 channels forming the spectrum of the colors associated independently to each pixel and used to reconstruct an image in grey tones. On the other hand, attaching some extra lines to the algorithm code, it is possible to rejoin the percentage dose values in small groups segmented to different depths. As an example, the values within 1–10% interval are assigned to a unique grey tone. Once this process is continuously carried out up to 91–100% interval, the image from Figure 7(a) could be obtained. The limiting lines at the borders within different tones represent the isodose curves, which are important to determine the optimum way to irradiate a tumor during a simulation of a therapeutic treatment for a patient.

#### 2.3.2. Visualization in Color HSI

The visual reconstruction of the values PD_new_ in a color image is appropriate to expose the regions with more or less radiation (hot or cold). One of the reconstruction models commonly used is the HSI (hue, saturation, and intensity) for the transformation based on numerical value ranges in terms of their components. Such definition is not standardized and other abbreviations can be used to obtain the same sense.

The reconstruction starts with the matrix obtained from (4) and to construct the image the following transformation equations are established for the colored space HSI [12]; it means that a transformation from the color RGB (4) to the color HSI is necessary by using transformation equations (5) to (8):

(5) (IV1V2)=[13  13  1316  16  1612  12  0]  (RGB),H=tan−1(V1V2),S=V12+V22.

Three values (*I*, *V*_1_, *V*_2_) for each pixel are obtained as indicated by (6); furthermore, a vectorial product was obtained from the coefficients matrix as indicated in (7):

(6) (ISsin⁡(H)Scos⁡⁡(H)).

A value group is obtained for each pixel within the RGB color space [12]:

(7) (RGB)=[13  13  1316  16  1612  12  0](ISsin⁡(H)Scos⁡⁡(H)).

**Figure 8:**
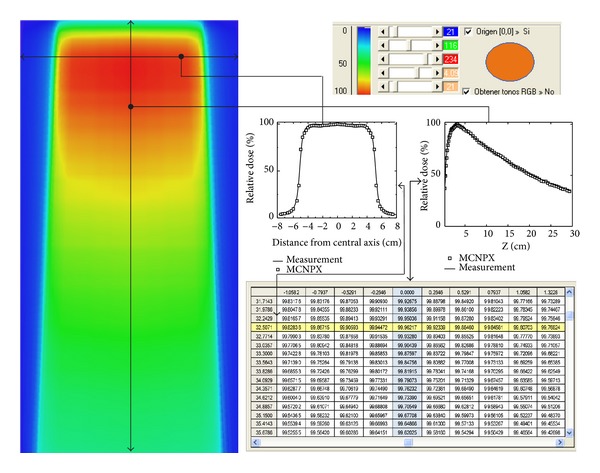
PD_new_ percentage values matrix for the HSL colored scale.

Finally the RGB values obtained from (7) were transformed to get the HSI values from (8). Such values were the ones needed to reconstruct the images within the HSI color space, as illustrated in (Figure 8)

(8) I=R+G+B3,H=G−B3I−3B,  S=I−BI si  B<R,G,H=B−R3I−3R+1,  S=I−RI si  R<B,G,H=R−G3I−3R+2,  S=I−GI si  G<R,B.

In the left side a colored red-orange panel is observed corresponding to the region of higher absorbed dose, reproduced from the percentage values matrix from the right side. The blue color column in the matrix represents the PDD and the yellow color line the data profile initially feeding the algorithm. The PDD and the absorbed dose profile correspond to the same group of values used to reproduce (Figure 7(a)). In the colored image the relationship between degradation of the new PD_new_ values according to the tones and the range of colors (red-orange, yellow, green (light-dark), and blue (light-dark)) is also observed. The region in the red-orange color corresponds to the maximum intensity and within the range of 80–100%. The yellow color region corresponds to the 70–80% while the green color region to the 25–70% and the light-dark blue color to the 0–25% one.

A summary of the terminology used is obtained in Abbreviation Section.

## 3. Conclusions

A dosimetric algorithm was codified and implemented to reproduce the absorbed doses induced by a radiation beam over a water phantom, which simulates human tissue. It was fed with data considering the absorbed doses PDD and their profiles with maximum percentage values located in the build-up region.

The software developed allows processing two groups of data (PDD and absorbed dose profiles) obtained initially by Monte Carlo techniques. A good agreement was found between the PD_new_ values reproduced by the algorithm and the simulated with MCNPX from the primary photon beam that is emitted from the head of the 18 MV LINAC.

Comparing PD_new_ percentage values reproduced from different depth profiles, a convenient agreement was found, even for the build-up region, where commercial algorithms used for planning are critical [13]. However, some discrepancies in the absorbed doses border at the water phantom were observed due to low photon interaction which transfers their energy to the electrons in the phantom's surface.

The main advantage for using the dosimetric algorithm to obtain the PD_new_ values is the optimization of the computation time (few seconds) for absorbed dose percentages, considering that acceptable values obtained by the Monte Carlo simulation take 30 days. There is also an advantage on the reproduction of the absorbed doses in the whole volume.

The uncertainty associated in the absorbed dose values calculated by Monte Carlo was below 1% in all cases, when compared with the PDD and measured profile, while the uncertainty obtained between PD_new_ values from the profiles obtained with the dosimetric algorithm and the ones obtained from Monte Carlo at different depths were below 2% in all cases within the beam field. Although it is not important the error at the penumbra region is below 5%.

Considering image reconstruction, either for grey tones or for colored HSL or for RGB [12, 14], it is very useful to analyze the way the radiation beam interacts with the phantom using the percentage values generated by the dosimetric algorithm pixel by pixel.

## Abbreviations

*V*_PPD_*i*__: Profile percentage values *i*th, voxel dose profile obtained by MCNPX
*V*_PDD_*j*__: Percentage depth dose values *i*th, voxel dose at a depth obtained by MCNPX
*V*_pix_*C*_PPD_*j*__: Interpolated value of the dose profile rate between *V*_PPD_*i*+1__ and *V*_PPD_*i*__
*V*_pix_*C*_PDD_*k*__: Interpolated value of the dose at a depth rate between *V*_PDD_*i*+1__ and *V*_PDD_*i*__
*θ*: Divergent radiation beam angle formed from the beam axis
PD_new_(*i*, *j*): Percentage depth dose calculated from (*V*_pix_ *C*_PPD_*j*__, *V*_pix_ *C*_PDD_*k*__)
PD_new_(*x*_*i*_, *y*_*j*_, *z*_*k*_): Percentage depth dose calculated at any point (*x*, *y*, *z*)
*I*(*i*, *j*): Matrix representation of the percentages of the dose converted to image
*H*, *S*, *I*: Color model defined in terms of its components (hue, saturation, and intensity)
*I*, *V*_1_, *V*_2_: Intensity and intermediate values 1 and 2
*R*, *G*, *B*: Representative color model combining the three primary colors (red, green, and blue).
